# Enabling 3D bioprinting of cell-laden pure collagen scaffolds via tannic acid supporting bath

**DOI:** 10.3389/fbioe.2024.1434435

**Published:** 2024-09-04

**Authors:** Sara Palladino, Francesco Copes, Pascale Chevallier, Gabriele Candiani, Diego Mantovani

**Affiliations:** ^1^ Laboratory for Biomaterials and Bioengineering, CRC-Tier I, Department of Mining, Metallurgy and Materials Engineering and Regenerative Medicine CHU de Québec, Laval University, Quebec City, QC, Canada; ^2^ GenT_LΛB, Department of Chemistry, Materials and Chemical Engineering ‘G. Natta’, Politecnico di Milano, Milan, Italy

**Keywords:** collagen, tannic acid, extrusion bioprinting, bioink, 3D printing supporting bath

## Abstract

The fabrication of cell-laden biomimetic scaffolds represents a pillar of tissue engineering and regenerative medicine (TERM) strategies, and collagen is the gold standard matrix for cells to be. In the recent years, extrusion 3D bioprinting introduced new possibilities to increase collagen scaffold performances thanks to the precision, reproducibility, and spatial control. However, the design of pure collagen bioinks represents a challenge, due to the low storage modulus and the long gelation time, which strongly impede the extrusion of a collagen filament and the retention of the desired shape post-printing. In this study, the tannic acid-mediated crosslinking of the outer layer of collagen is proposed as strategy to enable collagen filament extrusion. For this purpose, a tannic acid solution has been used as supporting bath to act exclusively as external crosslinker during the printing process, while allowing the pH- and temperature-driven formation of collagen fibers within the core. Collagen hydrogels (concentration 2–6 mg/mL) were extruded in tannic acid solutions (concentration 5–20 mg/mL). Results proved that external interaction of collagen with tannic acid during 3D printing enables filament extrusion without affecting the bulk properties of the scaffold. The temporary collagen-tannic acid interaction resulted in the formation of a membrane-like external layer that protected the core, where collagen could freely arrange in fibers. The precision of the printed shapes was affected by both tannic acid concentration and needle diameter and can thus be tuned. Altogether, results shown in this study proved that tannic acid bath enables collagen bioprinting, preserves collagen morphology, and allows the manufacture of a cell-laden pure collagen scaffold.

## 1 Introduction

In the context of Tissue Engineering and Regenerative Medicine (TERM), 3D bioprinting has sparked the interest of researchers in recent years (Murphy and Atala, 2014; Li et al., 2016; Mota et al., 2020). This additive manufacturing technique uses bioinks, defined as formulations of biomaterials and cells, to print tissue-like three-dimensional (3D) structures. 3D bioprinting introduced advanced features in the field, including micrometric precision, high reproducibility, and spatial control of biomaterial and cell deposition. Altogether, these features allow to process complex geometry constructs, unachievable with traditional fabrication techniques (Mota et al., 2020). Among the different 3D bioprinting approaches, extrusion-based bioprinting is one of the most used techniques, and it is especially suitable for hydrogels, widely used in TERM. Among the hydrogels for TERM applications, natural polymer-based ones are the most studied. Collagen represents, among them, the gold standard for scaffold processing, thanks to its remarkable biological properties, and its abundance in the human body, both in soft and hard tissues (Wang et al., 2023; Dong and Lv, 2016; Rezvani Ghomi et al., 2021). Moreover, being the main component of the extracellular matrix (ECM), collagen provides a physiological environment to the embedded cells, which will find molecular cues to guide and support their metabolism and functions. The combination of collagen with cells in TERM has been widely investigated for *in vitro* and *in vivo* studies, as well as for clinical applications (Wang et al., 2023; Lin et al., 2019), and more recently for the development of 3D printable bioinks. However, collagen is also known to lack adequate mechanical and rheological properties, especially for 3D printing (Lee et al., 2021; Osidak et al., 2020). On one hand, the low storage modulus and the weak mechanical properties strongly limit the extrusion of a defined filament and the structural fidelity post-printing, thus leading to imprecise and uncontrollable material deposition. On the other hand, once collagen assembles into its characteristic fiber structure, the rheological properties hamper the needle extrusion, thus leading to needle clogging and limiting the whole printing process. This leads to the critical and urgent need to develop effective strategies for 3D bioprinting specific to collagen-based structures. The ideal solutions must provide structural fidelity to the printed scaffold without modifying collagen structure and properties. Despite the significant advancements in this field, approaches for pure collagen bioinks are rather limited due to the highly challenging manipulation of collagen hydrogels.

In this context, the improvement of collagen crosslinking has been rather investigated, using thermal (Moncal et al., 2019), pH (Campos et al., 2015) or chemical crosslinkers (Kim et al., 2016). Among the chemical crosslinkers, tannic acid represents an interesting candidate. Tannic acid (TA) belongs to the polyphenol’s family, plant-derived organic compounds already extensively reported in the literature for biomedical applications due to their therapeutic properties (Baldwin and Booth, 2022; Gao et al., 2021). In fact, its anticancer (Youness et al., 2021; Cass and Burg, 2012), antioxidant (Perumal et al., 2019), antiviral and antibacterial (Kaczmarek, 2020), and anti-inflammatory properties (Yeo et al., 2020) have been widely reported and are rather acknowledged worldwide. TA carboxyl and hydroxyl groups ease its interaction with proteins and biomolecules. Interestingly, TA showed a high affinity for proline, an amino acid abundant in the collagen structure (Velmurugan et al., 2014; Gauza-Włodarczyk et al., 2017). For this reason, blending TA and collagen for scaffolds (Cass and Burg, 2012; Velmurugan et al., 2014; Kaczmarek et al., 2018), beads (Baldwin et al., 2020), films (Sionkowska et al., 2023), and bioinks (Yeo and Kim, 2017) has been already reported. However, the potential toxicity of TA could represent a drawback for its use. TA toxicity has been investigated and is controversial. While some *in vitro* and *in vivo* studies report no or very limited effects on viability and behavior of osteosarcoma cells (SaOS-2) and fibroblasts (3T3) when TA is combined with natural polymers (Kaczmarek et al., 2018; Kaczmarek et al., 2017; Shah et al., 2019), some others report detrimental effects. In fact, there is a correlation between TA concentration and biological performance. While low TA concentrations in combination with collagen have shown no major effect on mouse osteoblasts (MC3T3-E1) viability (Lee et al., 2018), other studies reported a dramatic decrease, especially for human liposarcoma cells (SW872) attachment and viability, when increasing the TA concentration (Baldwin et al., 2021). Moreover, despite these promising results, TA represents one of the main constituents of the developed hydrogels, therefore not allowing the printing of pure collagen constructs. Differently, this study seeks to avoid the presence of any secondary permanent component within the final collagen scaffold. In this direction, the use of TA for collagen crosslinking, in the form of an external and temporary bath rather than as permanent hydrogel component, represents a viable and novel solution to improve collagen printability without leaving traces post-printing.

The use of support baths has already been investigated in 3D bioprinting. These baths, usually composed of sacrificial gels (Hinton et al., 2015; Isaacson et al., 2018; Allencherry et al., 2022), provide a physical support, thus preventing structural collapse and improving shape fidelity of the bioink. TA solution can be used as support bath to provide, instead of a physical support, a chemical support by crosslinking collagen during the printing and be removed once the procedure is over. Compared to the complexity of using sacrificial gels for physical support, this strategy can provide a one-step, easy to implement, method for pure collagen bioprinting.

In this scenario, this study aimed to enable a single-step process for pure collagen bioprinting using an external temporary TA-supporting bath. The effect of the external and temporary interaction between TA and collagen was investigated in terms of molecular structure, morphology, and rheological properties. Additionally, both the effect of collagen and TA concentrations were investigated. Finally, the biological performances were assessed.

## 2 Materials and methods

### 2.1 Materials

Neonatal human dermal fibroblasts (nHDFs), high glucose Dulbecco’s Modified Eagle Medium (DMEM), Penicillin/Streptomycin, Hepes and fetal bovine serum (FBS) were purchased from ThermoFisher Scientific (United States). TA and glutaraldehyde were purchased from Sigma-Aldrich (United States). Rat tails were obtained free of charge from a certified animal facility. 3D printing consumables (needles, cartridges, pistons, stoppers) were purchased from Nordson Corporation (United States).

### 2.2 Collagen type I extraction and solubilization

Collagen type I was extracted from rat tail tendons, according to the protocol previously published by our group (Rajan et al., 2007). Following extraction, two different collagen stock solutions were prepared: collagen was suspended in 0.02 N acetic acid at 4 mg/mL and 8 mg/mL. Due to the high thickness of the 8 mg/mL solution, the suspension was kept at 4 C for 5 days and manually inverted for 30 s 3 times per day to achieve complete dissolution, as proposed by Cross et al. (2010). Both stock solutions were sterilized using Spectrapor dialysis bags (Mw 6–8 kDa) against sterile 0.02 N acetic acid at 4 C while stirring. The obtained collagen solutions were stored at 4 C.

### 2.3 Collagen hydrogels preparation

Collagen hydrogels at 2 mg/mL, 4 mg/mL, and 6 mg/mL were prepared in this study. Henceforward, they will be referred to as COL2, COL4, and COL6, respectively. Collagen stock solution at 4 mg/mL was used for the preparation of COL2 hydrogels, while the stock solution at 8 mg/mL was used for both COL4 and COL6. For hydrogels preparation, three components were mixed on ice: collagen stock solution (Sol_stock_) was mixed with a buffer solution (Sol_buffer_), specifically prepared depending on the final collagen concentration. Once a homogeneous solution was obtained, the appropriate amount of cell-free or cell-containing DMEM (Sol_medium_), depending on the test to be performed, was added to the solution. Following preparation, hydrogels were incubated at 37 C for 30 min. For COL2 and COL4 hydrogels preparation, the volume ratio of Sol_stock_: Sol_buffer_: Sol_medium_ was kept at 50%: 25%: 25%. For COL6, the ratio was modified to 75%: 15%: 10% to obtain the desired final concentration. The abovementioned Sol_buffer_ was invariably based on concentrated DMEM, with the addition of 4 μmol Hepes and 3 μmol NaOH per 100 μL of Sol_stock_. Detailed volumes for collagen hydrogel preparation are listed in Table 1. This protocol was an adaptation of our previous works using higher collagen concentrations (Rajan et al., 2007; Cross et al., 2010).

**TABLE 1:** Detailed composition of the collagen hydrogels at different concentrations used in this study.

		COL 2 mg/mL		COL 4 mg/mL		COL 6 mg/mL	
		%vol	Vol for 1 mL hydrogel	%vol	Vol for 1 mL hydrogel	%vol	Vol for 1 mL hydrogel
Collagen stock solution		50%	500 μL (at 4 mg/mL)	50%	500 μL (at 8 mg/mL)	75%	750 μL (at 8 mg/mL)
Collagen buffer solution	Concentrated DMEM	25%	215 μL (5X DMEM)	25%	215 μL (5X DMEM)	15%	111 μL (10X DMEM)
	Hepes		20 μL (1 M Hepes)		20 μL (1 M Hepes)		30 μL (1 M Hepes)
	NaOH		15 μL (1 M NaOH)		15 μL (1 M NaOH)		9 μL (2.5 M NaOH)
DMEM (with or without cells)		25%	250 μL	25%	250 μL	10%	100 μL

### 2.4 Collagen hydrogels preparation in tannic acid

Three different TA concentrations were studied: 5 mg/mL, 10 mg/mL, and 20 mg/mL, henceforward referred to as TA5, TA10 and TA20, respectively. For all the morphological and structural analyses, the collagen solution was manually pipetted into a liquid TA bath, which was immediately removed afterward. For all the 3D (bio) printing tests, the collagen solution was immediately loaded into a printing cartridge, and extruded in a 6-well plate, previously filled with the TA supporting bath. The liquid bath was manually removed at the end of the printing process. In both cases, following TA removal, samples were transferred at 37 C for 30 min.

### 2.5 Inversion test

As a proof-of-concept, COL4 was manually pipetted into a glass vial containing a TA10 solution. The vial was inverted at 0, 5, 10, and 30 min, and pictures were taken at each time point. In the control vial, COL4 was prepared without contact with the TA. Between time points, vials were incubated at 37°C.

### 2.6 Scanning electron microscopy (SEM)

For SEM analysis, 500 μL collagen hydrogels were prepared by manual pipetting into 24-well plates. TA bath volume was set at 500 μL/well, and empty wells were used as controls. Samples were washed in PBS, fixed in 5% v/v glutaraldehyde solution overnight, then washed in deionized H_2_O (dH_2_O) and dehydrated in alcohol series (30%–50%–70%–90%–100%) (Xu et al., 2021; Martinez-Garcia et al., 2022). Following incubation overnight in 100% ethanol solution, samples were washed again in dH_2_O and frozen at −80°C. Finally, samples were freeze-dried. For cross-section, dry samples were cut using a scalpel blade, and placed vertically on the sample holders. All samples were coated twice with gold-palladium using a Polaron SC500 Sputter Coater (Quorum, UK). Samples images were acquired using the Quanta 250 SEM system (FEI Company Inc. Thermo-Fisher Scientific, United States) with an acceleration voltage of 15 kV.

### 2.7 Fourier transform infrared spectroscopy (FTIR)

Sample preparation for chemical structure analysis was carried out following the same protocol for SEM analysis, with the exclusion of the coating step (cfr 2.6). Samples were analyzed by Attenuated Total Reflectance-Fourier Transform Infrared (ATR-FTIR) spectroscopy, using a commercial spectrometer (Agilent Cary 660 FTIR, Agilent Technologies, United States), equipped with a deuterated L-Alanine-doped triglycine sulfate (DLa-TGS) detector and a Ge-coated KBr beam splitter. Spectra were recorded in absorbance mode with a 4 cm^−1^ resolution, ranging from 4,000 to 400 cm^−1^. The number of scans per spectrum was set to 64.

### 2.8 Rheological characterization and sample volume stability

Rheological characterization was performed using the non-destructive and contactless measurement system ElastoSens^TM^Bio (Rheolution, Canada). For each condition, 4 mL collagen solution was pipetted into a sample holder containing 1.5 mL TA solution, which was gently removed following sample preparation. Sample elasticity was measured in stiff mode for 30 min. The temperature in the testing chamber was kept at 37 C throughout the test duration. Storage and elastic modulus were measured with a 30-s frequency. Sample height, before and after 30 min of incubation, was measured using the laser measurement system of the abovementioned instrument.

### 2.9 Cell culture

Fibroblasts (nHDFs) were cultured in DMEM supplemented with 10% FBS and 1% Penicillin/Streptomycin, henceforward referred to as cDMEM. Cell culture was carried out at 37 C, under 5% CO_2_ supply, in a humidified atmosphere, as in *vitro* standard cell culture conditions. Cells were subcultured at 80% of confluence and the culture medium was replaced every other day. For cell-laden collagen preparation, the appropriate number of cells was centrifuged and resuspended in cDMEM before bioprinting. Only cells between passages 6 and 8 were used for the experiments.

### 2.10 3D printing and bioprinting

All 3D printing and bioprinting tests were carried out using a screw-based extrusion bioprinter (Regemat V1, Regemat, Spain), with flow speed 15 mm s^−1^. Cylindrical needles of 15G (1.36 mm), 18G (0.84 mm), and 20G (0.61 mm) internal diameter, and 12.7 mm length were used during the printing parameter optimization. Collagen (bio) ink was prepared, either cell-free or containing 1 × 10^6^ cells/mL, by mixing Sol_stock_, Sol_buffer_, and Sol_medium_ accordingly to the protocol described above (cfr. 2.3). This procedure was carried out on ice to prevent collagen fibrillogenesis. Immediately after mixing the three components on ice, the collagen solution was loaded into the printing cartridge and the printing process was started. Given the collagen thermos-responsive behavior, the whole printing process was carried out within 3 min from cartridge loading, at room temperature. A 6-well plate containing 1.5 mL/well of tannic acid was used as printing support. For all characterizations, a line-shaped construct (2 cm in length, 0.8 cm in height) was printed. Following 3D (bio) printing, TA was removed, and pictures were taken to assess the extrusion and shape fidelity. The thickness of the extruded lines was measured from the pictures both at the two extremities and at the middle section. The offset of the measured thickness from the needle diameter was calculated to analyze the difference between the theoretical and the 3D printed strut. Following image acquisition, cDMEM was immediately added to the all samples containing cells to provide a suitable environment for cell survival.

### 2.11 Biological characterization post-bioprinting

Cell viability within the 3D bioprinted samples was evaluated using a modified live/dead staining. On days 1, 3, and 7, samples were washed in PBS and incubated for 20 min at room temperature with the staining solution. Calcein AM (1 μM) was used to stain the cytoplasm of live cells. DAPI (0.625 μg/mL) was used, without any cell permeabilization step, to stain dead cells nuclei, as their damaged membrane allows for dye penetration, as previously reported (Zuncheddu et al., 2021; Sturm et al., 2021; Palladino et al., 2023). Given the low concentration and the short incubation time, it can be reasonably assumed that DAPI stains exclusively dead cells. Stained samples were imaged using an LSM800 confocal microscope (Carl Zeiss, Germany). For a quantification of the live and dead cells, 2D images (n = 3) were taken for each sample, and the percentage of green- and blue-stained cells was evaluated. Being a destructive analysis, different samples were used at each time point, for each condition.

### 2.12 Statistical analysis

Statistical analysis was performed using Excel (Microsoft, United States). Statistical significance was evaluated via two tails Student’s t-test for homoscedastic samples. Results were considered statistically significant for *p*-values <0.05. Data are shown as mean ± standard deviation.

## 3 Results

### 3.1 Morphological, structural, and rheological characterization

**FIGURE 1:**
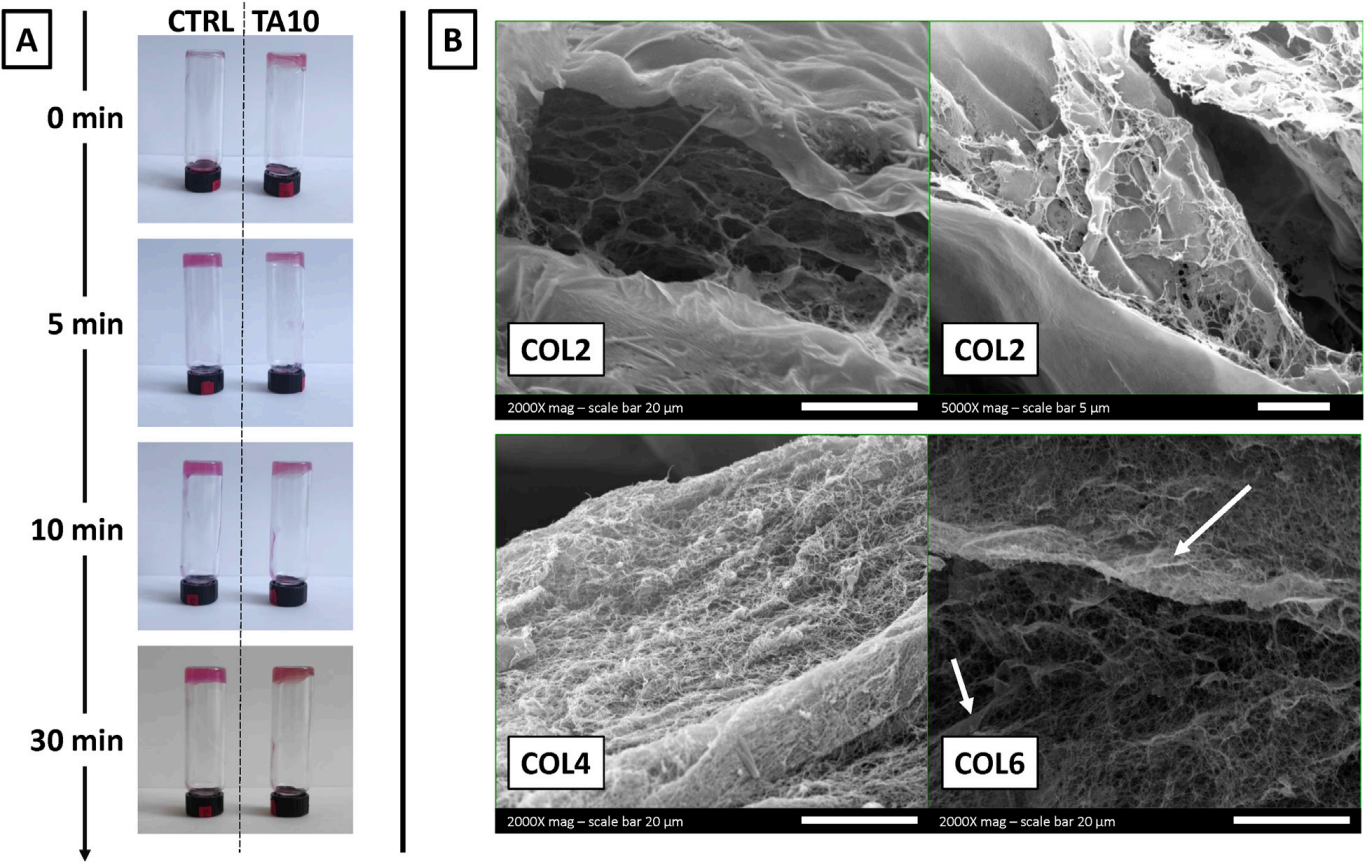
**(A)** Inversion test performed on COL4 without TA (control condition) and with 10 mg/mL TA (TA10, experimental condition) **(B)** SEM images of COL2, COL4, and COL6 combined with TA10. The inversion test proves, qualitatively and macroscopically, the TA-mediated crosslinking of collagen at time 0. SEM images prove at the microscopic level that the ability of collagen to assemble into fibers is preserved after the interaction with TA. However, SEM images also suggest that at a low concentration (COL2), TA hampers the organization into fibers.

Figure 1A shows macroscopic pictures of the inversion test, taken at each time point after inverting the vials. The test was performed exclusively on COL4, as a representative condition, without (left) and with (right) 10 mg/mL of TA. At time 0, immediately after sample preparation, collagen prepared in TA was not flowing along the vial walls, unlike what was observed for the control. Starting 5 min post-incubation at 37°C, both samples showed similar behavior throughout the remaining 25 min of observation. Overall, these results showed a difference between the gel-like structure of COL4-TA10 and the COL4 control during the first few minutes of the test. No other difference (i.e., color) was macroscopically apparent between the two conditions during the whole test.

Figure 1B shows the morphology observed via SEM for three different collagen concentrations (COL2, COL4, and COL6) combined with 10 mg/mL TA. For both COL4 and COL6, it is possible to observe a network of fibers, which represents the typical organization of collagen *in vivo* and *in vitro* (following hydrogel formation). Additionally, with COL6, some fiber agglomerations could be observed, differently from the COL4 condition. Conversely, with COL2 no fibrillar structure could be observed, as further confirmed at higher magnification. During sample preparation, hydrogel formation was macroscopically and qualitatively observed for all conditions, including COL2. However, SEM results could not confirm the presence of collagen fibers at 2 mg/mL. Overall, microscopy results confirmed the presence of the typical collagen fibers for a minimum concentration of 4 mg/mL.

**FIGURE 2:**
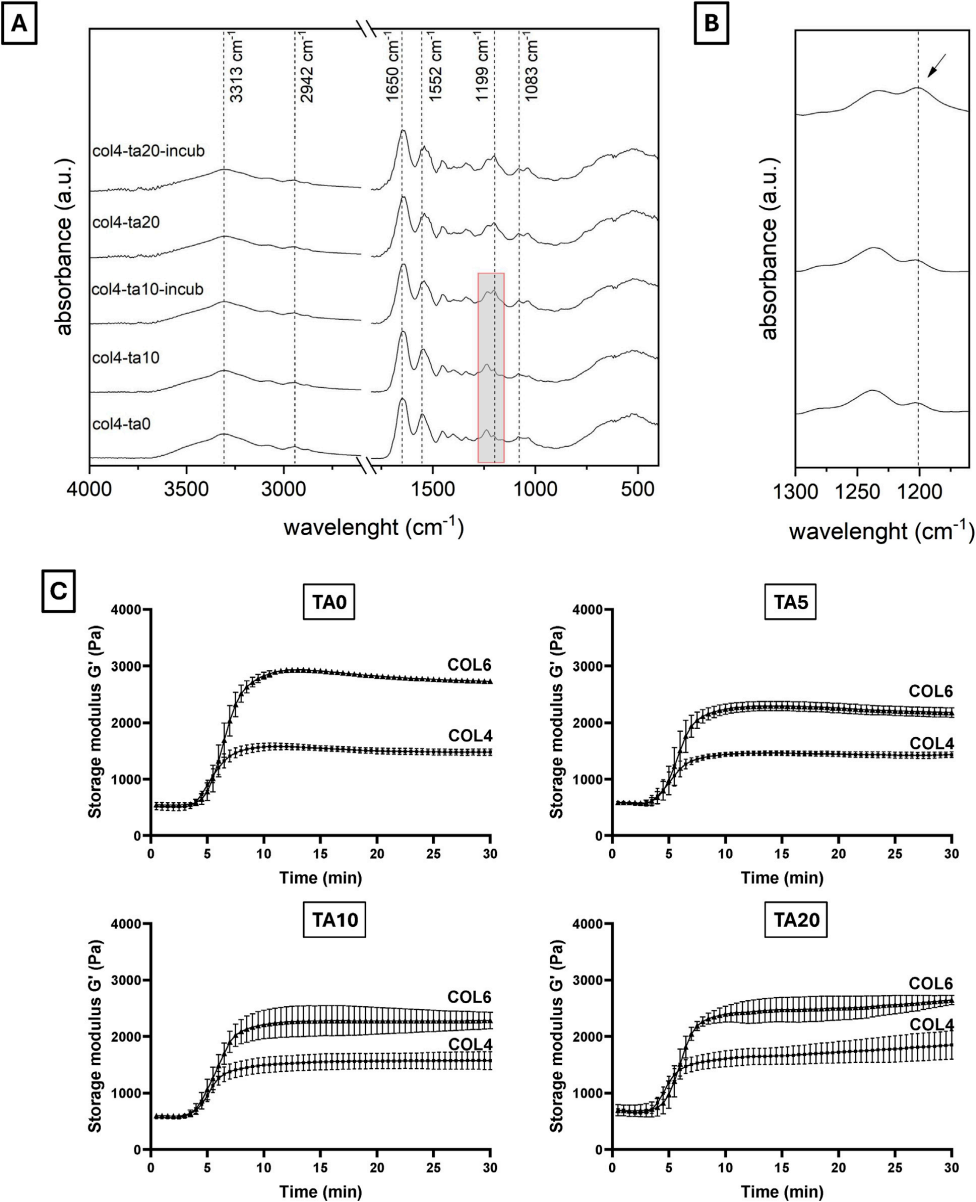
**(A, B)** FTIR spectra and **(C)** storage modulus of different COL-TA combinations. FTIR spectra show the characteristics collagen peaks for all tested conditions. A shift of the 1,650 cm^−1^ and 1,552 cm^−1^ peaks was observed for the TA-treated samples. Furthermore, the TA-characteristic peak at 1,199 cm^−1^ appeared for TA10 after incubation (see enlarged spectrum). **(C)** Rheological properties significantly increase by increasing the collagen concentration. They are not altered by the use of TA on the external layer.

FTIR analysis investigated the molecular structure of different collagen and TA combinations (Figure 2). FTIR spectra are shown in Figure 2A. All spectra, independently from TA concentration and incubation time, show the characteristic collagen peaks, corresponding to amide I (1,650 cm^−1^), amide II (1,552 cm^−1^), amide III (1,238 cm^−1^), amide A or ʋ_NH_ (3,313 cm^−1^), and amide B or ʋ_CH2_ (2,942 cm^−1^) (Nashchekina et al., 2020; Riaz et al., 2018; Li et al., 2023). Additionally, the peak at 1,083 cm^−1^, corresponding to the δ_C-O_, identifies the presence of collagen fibrils within the sample (Nashchekina et al., 2020), as previously observed in SEM images. A significative shift (6–12 cm^−1^) was observed for the amide I and amide II peaks when comparing all samples prepared with TA to the COL4 control (Table 2), thus testifying an interaction/crosslinking in the presence of TA. Furthermore, a change to the peak at 1,238 cm^−1^ was observed in most conditions with TA, as shown in Table 2: no significant difference could be observed between COL4-TA10 and the COL4-TA0 control, while a peak at 1,199 cm^−1^, corresponding to TA (Chevallier et al., 2023), appeared on the spectrum when TA10 was incubated for 30 min. This additional peak was also observed in the COL4-TA20 spectra, both non-incubated and incubated. Between COL4-TA10-incubated, COL4-TA20, and COL4-TA20-incubated no significant difference could be observed. No other characteristic peaks nor significant shifts were observed in the spectra.

**TABLE 2:** Description, wavelength, and shifts of significant peaks observed in the FTIR spectra shown in Figures 2A, B.

Absorption peak	Wavenumber (cm^−1^)	Significant shifts
Amide I	1,650	Shift of 6 cm^−1^ for all TA-treated conditions
Amide II	1,552	Shift of 12 cm^−1^ for all TA-treated conditions
Amide III	1,238	Shift of 6 cm^−1^ for COL4-TA10-incub Shift of 12 cm^−1^ for COL4-TA20 and COL4-TA20-incub
Amide A	3,313	-
Amide B	2,942	-
Fibrillar collagen	1,083	-
Tannic acid	1,199	Appears in COL4-TA10-incub, COL4-TA20 and COL4-TA20-incub

Both COL4 and COL6 were studied in terms of rheological properties with different concentrations of TA. Given the abovementioned SEM results, COL2 rheological results are not shown. Figure 2C shows the storage modulus (G′) for all the COL-TA combinations (data for loss modulus not shown). For all conditions, curves can be divided into three main regions: an initial plateau (min 0–5), a transition phase (min 5–10), and a final plateau (min 10–30). Regardless of the TA concentration, data show a remarkable effect of the collagen concentration on the G′ value in the final plateau region (∼1,500 Pa for COL4 vs. ∼3,000 Pa for COL6). The presence of TA results in a slight decrease in G′ for all studied TA concentrations. This difference can be attributed to the preparation protocol for rheological characterization: the multiple pipetting into the TA solution in the sample holder caused the formation of some discontinuities within the hydrogel, which slightly affected the final measurements. Nevertheless, the starting values and the trends remained unaltered for all conditions. Interestingly, no difference among TA concentrations could be noticed (at the same collagen concentration).

### 3.2 Volume stability during hydrogel formation

**FIGURE 3:**
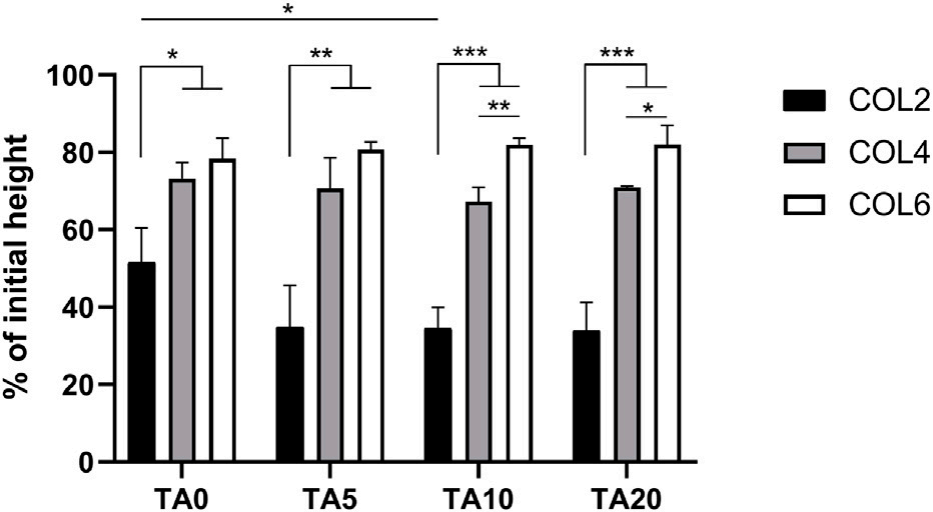
Volume stability in terms of height change following collagen fiber formation. COL4 and COL6 show higher volume retention capacity compared to COL2. No significant effect of the TA presence was observed. *p < 0.05, **p < 0.01, ***p < 0.001, n = 3.

The sample volume stability during collagen fiber formation (fibrillogenesis) was investigated. Sample height, before and after 30 min of incubation at 37°C, was recorded. Figure 3 shows the results in terms of percentage of the initial height for all the COL-TA combinations. Results show a significantly different behavior of COL2 compared to higher collagen concentrations. This difference could be observed both in the TA-free and in the TA-treated conditions (*p* < 0.05 COL2 vs. COL4 and COL6 for TA0; *p* < 0.01 COL2 vs. COL4 and COL6 for TA5; *p* < 0.001 COL2 vs. COL4 and COL6 for TA10 and TA20). At 2 mg/mL, with and without TA, collagen samples lose 50% minimum of the initial height during the hydrogel formation, due to the collagen rearrangement within the solution resulting in water expulsion. An average of 70% and 80% of the initial height was kept by the sample at COL4 and COL6, respectively. Specifically, the highest value for volume retention after incubation was observed for the COL6-TA10 and COL6-TA20 combinations (average 82% of initial height). Both for COL4 and COL6, statistical analysis revealed no significant changes caused by the contact with TA, at all tested TA concentrations.

### 3.3 Effect of TA on collagen morphology

**FIGURE 4:**
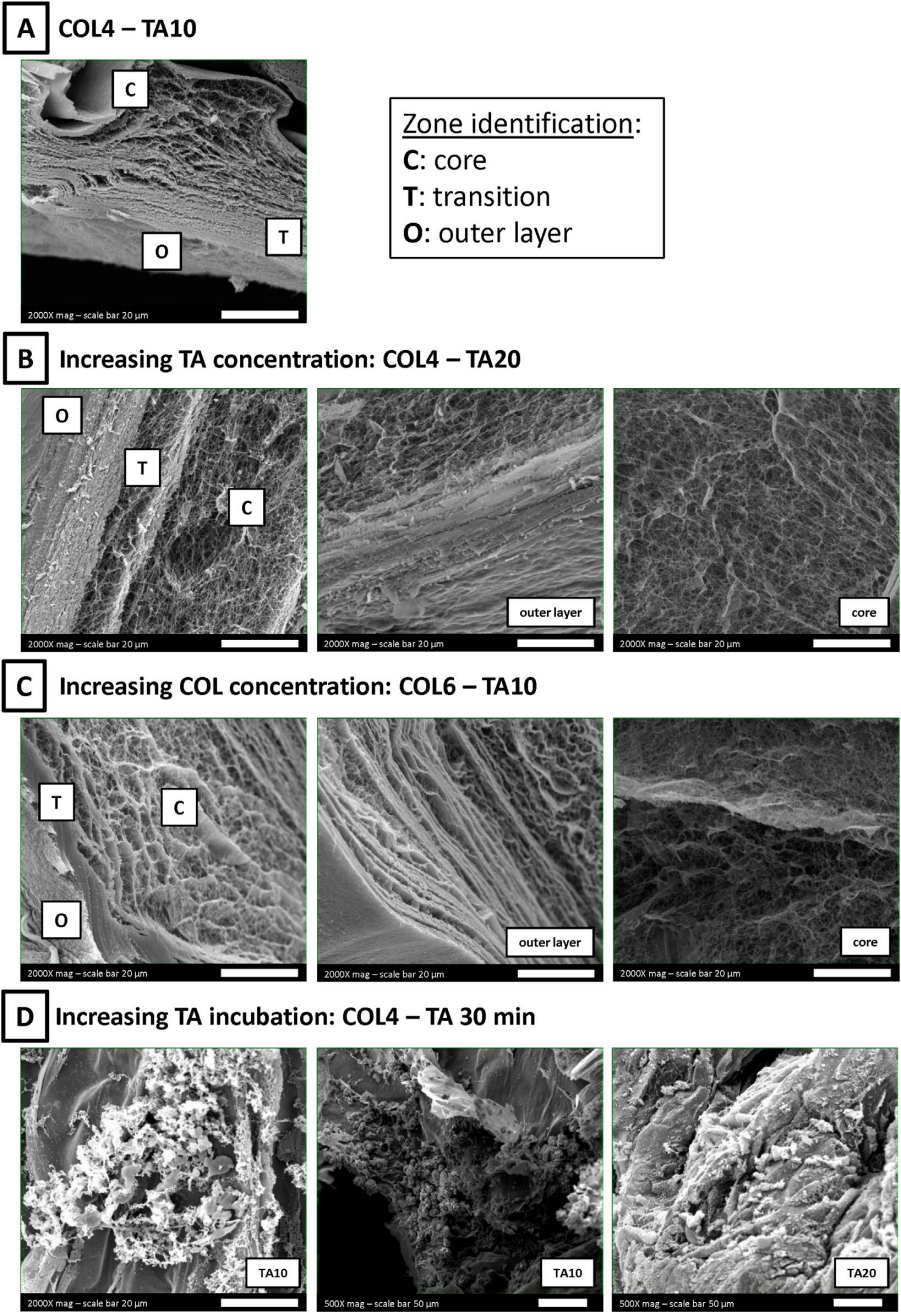
SEM images of COL-TA cross-sections. **(A)** A zone-dependent morphology was observed: (Murphy and Atala, 2014) an outer layer, more compact, generated by the interaction with TA; (Li et al., 2016) a core zone, rich in collagen fibers; (Mota et al., 2020) a transition zone between the two previous ones. **(B, C)** Each zone could be observed at each TA concentration at the collagen concentration tested. **(D)** On the contrary, the observed morphology was altered after longer collagen incubation with TA.

Additional SEM images were recorded to identify, if present, differences in collagen morphology between the shell and the core following the contact with TA. For this purpose, samples were cut, and the cross-section was investigated by SEM. Figure 4 shows representative images of different COL-TA combinations.

**FIGURE 5:**
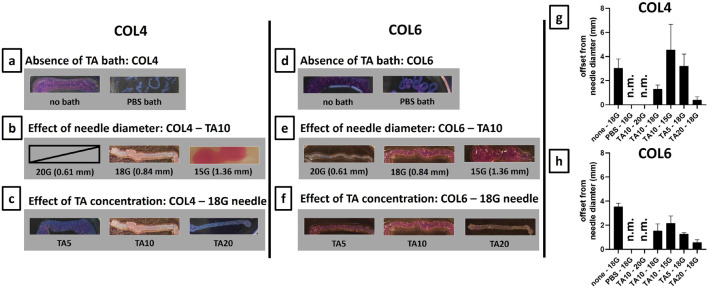
Optimization of 3D printing process on 20 mm lines for COL4 **(A–C)** and for COL6 **(D–F)**. For both collagen concentrations, the 18G needle gave the best results in terms of shape fidelity. On the contrary, extrudability was not possible/non-controllable with a 20G needle, and the line shape was lost with a 15G needle. Furthermore, for both COL4 and COL6, the increase in TA concentration resulted in improved shape fidelity. **(G, H)** Line thickness was measured both at the extremities and at the middle part of the samples. Results are expressed, as mean ± standard deviation, in terms of offset from the needle diameter (mm), and confirm the results obtained from the qualitative image analysis. For non-extrudable conditions and unshaped samples, results are indicated as non-measurable (n.m.). Image contrast was adjusted to allow better visualization of the printed samples.

**FIGURE 6:**
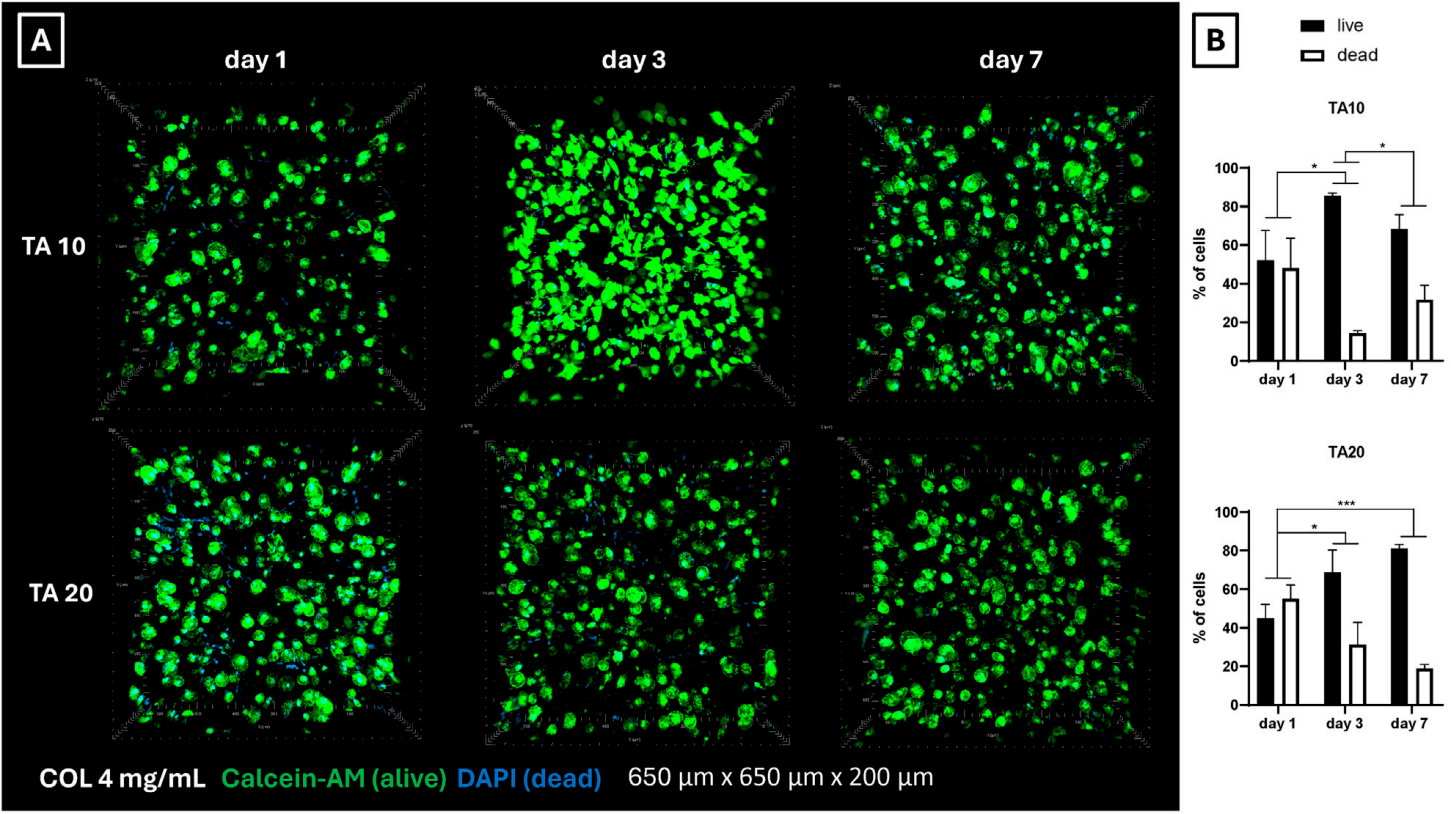
**(A)** Live/dead representative images of nHDFs post-bioprinting. On day 1 a considerable number of dead cell nuclei is visible for both conditions. However, cell viability significantly increases on days 3 and 7. Overall, a homogeneous cell distribution is observed, with no signs of cell agglomeration **(B)** Ratio between alive and dead cells post-bioprinting. Cell counting confirmed the mortality observed in the live/dead 3D images (Figure 6A). It was noticed a general tendency of increasing cell viability during *in vitro* culture for both conditions. *p < 0.05, ***p < 0.001, n = 3.

The combination COL4-TA10 was considered as the starting point for the analysis (Figure 4A). The cross-section revealed the presence of three definite areas (Murphy and Atala, 2014): a compact, “membrane-like”, outer layer (Li et al., 2016); a fibrillar core showing a visible fiber network, comparable to samples prepared without TA (Mota et al., 2020); a transition layer between the previous two, with mixed fibers and compact zones. The effect of TA concentration on each zone was investigated by doubling TA concentration from 10 mg/mL to 20 mg/mL. SEM images confirmed the presence of three areas, with characteristics comparable to the abovementioned ones (Figure 4B). Collagen fibers in the sample core were unaffected by the change in TA concentration during the preparation process. Collagen concentration effect on each zone was also investigated: the collagen concentration was increased from 4 mg/mL (Figure 4A) to 6 mg/mL (Figure 4C). SEM images still confirmed the presence of the three areas comparable to the abovementioned ones. The difference in collagen fiber organization (i.e., agglomeration) between COL4 and COL6 previously mentioned was confirmed in the sample core (Figure 4C, right). Finally, the time of TA incubation was increased to 30 min. Longer exposure to TA resulted in agglomerations and altered structures in the cross-section of these samples, in which it was possible to identify collagen fibers only in sporadic regions (Figure 4D). The same effect was observed following incubation with both TA10 and TA20.

### 3.4 Optimization of the 3D printing process

Figure 5 shows the images of the 3D printed constructs for both COL4 (Figures 5A–C) and COL6 (Figures 5D–F) and the measurements of the line thickness for all printed conditions (Figures 5G, H). In the 3D printing process, both the independent variables (viscosity and composition of the bioink) and the intrinsic variables (printing speed and needle diameter) strongly affect the shape fidelity. In this study, printing speed was set to 15 mm s^−1^ and therefore it was not considered in the optimization process. Figures 5A, D show the results in the absence of a TA bath, used as control conditions. In this case, the outcome depends exclusively on the viscosity and rheological properties of collagen. Results clearly show that in absence of the external TA-mediated crosslinking, collagen overflows or dissolves into the PBS bath and shape fidelity is poor.

Different needle diameters were tested to investigate the influence of the intrinsic variables on the printing outcome. Figures 5B, E show the results for COL4 and COL6, respectively. For both conditions, the hydrogel was not extrudable through a 20G needle: the outcome could not be measured due to the lack of printability. Although for COL6 a thin filament could be extruded (Figure 5E), the pressure applied for printing caused the water phase separation from the bioink inside the cartridge, thus leading to inhomogeneity. On the contrary, the extrusion was controllable, and the line shape was maintained post-printing with an 18G needle, both for COL4 and COL6 (Figures 5B, E). Finally, the increase in needle diameter to 15G resulted in no control on the filament extrusion, thus leading to an unshaped printed sample, for both collagen concentrations. Considering these results, an18G needle was selected as optimized intrinsic parameter for the TA-mediated approach. Following the optimization of the needle diameter, the effect of the composition was investigated: TA concentration was increased from 5 mg/mL to 20 mg/mL and the shape fidelity was assessed. Figures 5C, F show the results for COL4 and COL6, respectively. For COL4, a low TA concentration (TA5) led to shape fidelity loss, while a high concentration (TA20) significantly improved the outcome (Figure 5C). For COL6, no difference in shape fidelity was observed between TA5 and TA10, while a significant improvement was obtained with TA20, similar to the results for COL4 (Figure 5F). Overall, a similar trend was observed for COL4 and COL6: 18G was identified as the optimal needle diameter for both conditions and, in both cases, an increase in TA concentration led to improved shape fidelity post-printing. The measurements of the line thickness (Figures 5G, H) confirmed the abovementioned observations. The high difference from the theoretical needle diameter proves the need for a TA-mediated crosslinking and confirms 18G as the optimal needle diameter. Furthermore, both Figures 5G, H clearly indicate TA20 as the condition with the highest fidelity to the theoretical structure among the studied conditions. TA20 was confirmed as the optimal condition also in terms of homogeneity of the printed lines, as shown by the short standard deviation obtained for this condition.

### 3.5 3D bioprinting and biological performances

Biological performances were assessed on COL4 combined with TA10 and TA20, using an 18G needle, being these the optimized conditions found in the previous phase. Fibroblasts (nHDFs) were embedded in collagen and bioprinted in a line shape. Figure 6A shows the representative 3D images of the live/dead staining performed on the bioprinted samples 1, 3, and 7 days post-bioprinting. Live cells are stained in green, while dead cell nuclei are stained in blue. 2D images (data not shown) were used to quantify the number of cells at each time point. Figure 6B shows the average percentage of alive and dead cells at each time point and for each condition. Figure 6A shows a homogeneous distribution of cells within the observed volumes and the absence of clusters. At day 1 post-bioprinting, an important number of dead cells nuclei can be observed for both conditions, decreasing the cell viability at 45% for TA20 (Figure 6B). The percentage of dead cells is significantly decreasing during *in vitro* culture, as proved by results on day 3 and day 7. On day 7, the cell viability reaches values of 68% and 81% for TA10 and TA20, respectively. Overall, results show that short-time toxicity is present, which is considerably recovered during *in vitro* culture.

## 4 Discussion

The general aim of this study was to enable collagen bioprinting. Despite its outstanding biological properties, the use of collagen in additive manufacturing applications, such as 3D bioprinting, is still hampered due to the insufficient mechanical properties and long gelation time. In this study, different concentrations of collagen type I, ranging from 2 mg/mL to 6 mg/mL, have been used in the development of a bioink including human fibroblasts and bioprinted using a supporting bath comprising of TA at different concentrations (from 5 mg/mL to 20 mg/mL). Despite being often reported in literature as a main component of composite biomaterials, in this study TA role was exclusively as an external component, for the crosslinking of the external layer of collagen, to be removed immediately post-bioprinting.

**FIGURE 7:**
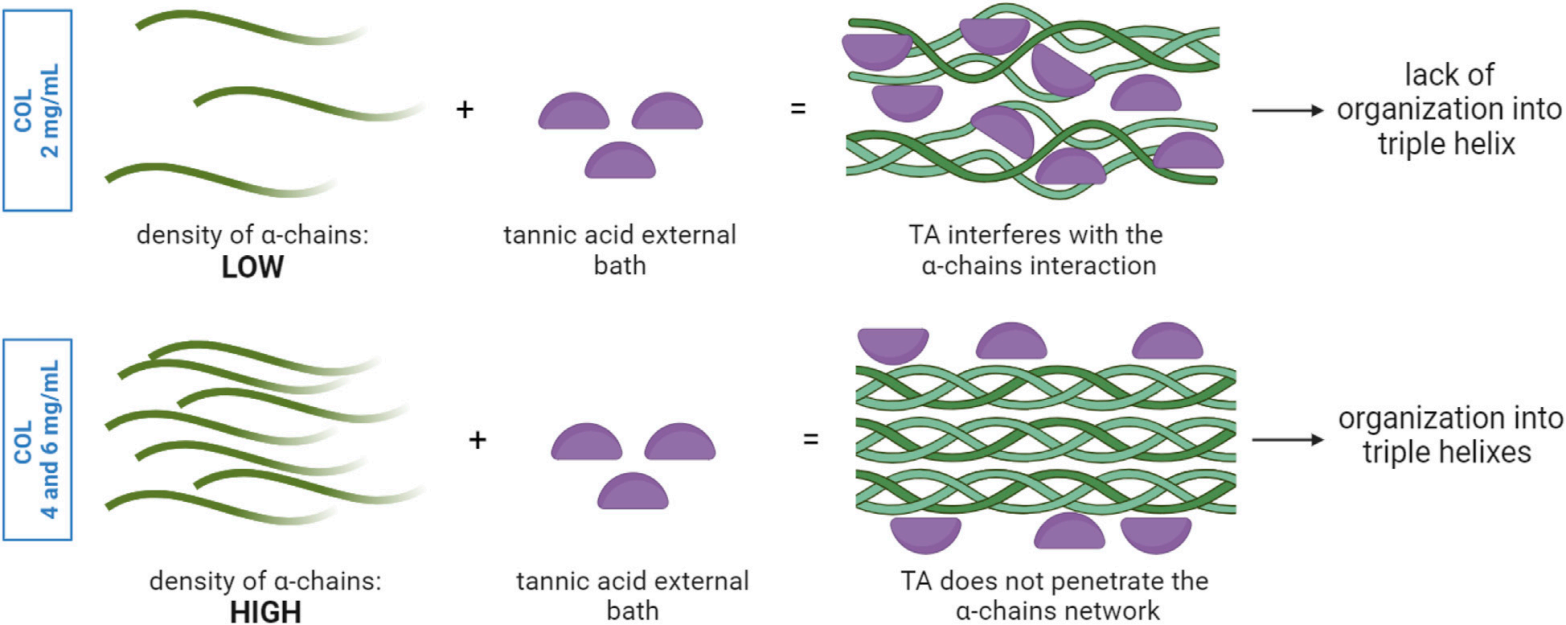
TA interaction with collagen α-chains network: the density of α-chains influences the diffusion of TA from the external bath into the core and thus the sites of interactions between TA and collagen. In a low-density α-chain network, TA molecules can penetrate and interact with the α-chains in the entire volume, thus altering the way α-chains can interact with each other. This could explain the lack of organization in triple helixes. In the presence of a higher density of α-chains, TA molecules lack sufficient time and space for diffusion and thus interact only with the α-chains at the outer layer, while the α-chains within the bulk organize in triple helixes.

Figure 1A shows the results from inversion tests. As it can be seen, already at time 0 the interaction between collagen and TA, despite being only external, is able to generate a macroscopically visible gel-like structure compared to the bare collagen. This shows the ability of TA to induce changes in collagen fiber formation. However, the interaction should not alter the bulk properties of the collagen scaffold. Collagen type I is known for its organization into fibers, distributed in a random and isotropic manner in *vitro* hydrogels (Achilli and Mantovani, 2010). SEM images of collagen-TA samples confirmed the presence of collagen fibers in the bulk both for COL4 and COL6 (Figure 1B), comparable to untreated collagen samples (Supplementary Figure S1). The presence of fiber agglomerates in COL6 is considered an effect of the increasing collagen concentration and not induced by TA, as these agglomerates are also visible in the non-treated COL6 as well (Supplementary Figure S1). Results for COL4 and COL6 suggest that TA did not penetrate the bulk of the collagen scaffold, as it has been shown before that TA treatment causes a change in collagen morphology (Natarajan et al., 2013). In contrast to the results of COL4 and COL6, no collagen fibers could be observed for TA-treated COL2 samples. Figure 1B shows a higher magnification SEM image as additional proof of the absence of fibers. Previous literature studies claimed that TA interaction with collagen, via hydrogen bonds and hydrophobic interactions, does not alter the integrity of the collagen triple helix (Velmurugan et al., 2014; Violeta et al., 2009). However, the results obtained in this study for COL2 are not consistent with previous literature findings. It is reasonable to hypothesize that the behavior herein observed is ascribed to collagen concentration, as illustrated in Figure 7.

The collagen solution at 2 mg/mL is characterized by a significantly higher water content and significantly lower density of α-chains compared to COL4 and COL6. Given the highly complex stereo structure of TA, the low collagen density within the solution could facilitate the TA molecules diffusion from the external layer to the core compared to highly dense solutions. Given that the interaction takes place during fibrillogenesis, namely, the reorganization of collagen into fibrils, the presence of TA molecules could physically hamper the pH- and temperature-driven process of fibril formation. The ratio between the number of TA molecules and of α-chains, combined with the steric effect and structural rigidity of TA, would thus influence the fibrollogenesis and explain the absence of collagen fibers. Despite this phenomenon not being previously reported, this hypothesis could reasonably support the obtained results. Further investigation is needed to corroborate the abovementioned theory. Despite the absence of fibers in the COL2 condition, SEM images of COL4 and COL6 were promising, as they showed the unaltered collagen morphology in the bulk of the samples. In order to corroborate the results obtained at the morphological level, FTIR analysis was performed on COL4 samples after contact with TA to evaluate its effect at a molecular level. Indeed, TA does not alter collagen chemical structure. As shown in Figure 2A, independently from TA concentration and incubation time, all conditions showed spectral characteristics comparable to the collagen control. The absence of major changes in the amide bands corroborates the hypothesis that the collagen fibrillar structure is maintained after interaction with TA, as mentioned above for the SEM images for the 4 mg/mL collagen concentration. Nonetheless, shifts in the amide I (6 cm^−1^) and amide II (12 cm^−1^) were observed, thus indicating an interaction between COL and TA. Similarly, Jastrzebska et al. (2006) reported amides I and II shifts in TA-treated pericardium tissues, rich in collagen, that could be ascribed to the formation of hydrogen bonds. Comparable shifts were also found by Sionkowska et al. (2023) after the TA-treatment of fish collagen films. Interestingly, a new absorption peak at 1,199 cm^−1^ was observed for the COL4-TA10-incubated, COL4-TA20, and COL4-TA20-incubated conditions (Figure 2B). This peak, as previously reported, is the C-O stretching from the TA (Chevallier et al., 2023). This suggests that the incubation of the collagen scaffold in TA, along with the increase in TA concentration, have a comparable effect in terms of residual ‘contamination’ of TA in the sample. Due to the depth of analysis of FTIR (approximately 1 μm), this observation is valid merely for the external layer of the samples. A higher TA concentration could be harder to wash away when removing the supporting bath, thus explaining the presence of the peak in the FTIR. Despite the proven interactions happening between TA and collagen, as shown by the FTIR results, the rheological analysis revealed no significative effect of TA on collagen scaffolds in terms of storage modulus (Figure 2C). In the literature, TA has already been reported as reinforcement for collagen scaffolds, and a significant increase in storage modulus and mechanical properties has been shown in literature (Yeo and Kim, 2017; Sarker et al., 2023; Wu et al., 2019). However, in the abovementioned studies TA is used as permanent constituent of the collagen-based formulation, thus directly impacting the bulk properties. On the contrary, in this study, TA was meant to exclusively interact with the external portion of the collagen hydrogel. Since the rheological analysis considers the entire sample volume, rather than the external surface, as analyzed via FTIR, we can conclude that the effect of TA, even at different concentrations, is limited to the outer portion of the collagen hydrogel, leaving the bulk storage modulus unaltered. On the other hand, the collagen concentration affects the bulk properties: there is indeed a correlation between the changes in concentration and the variations in storage modulus (G′). Figure 2C shows an increase in G′ in the plateau region from approximately 1,500 Pa of COL4 to approximately 3,000 Pa of COL6. The bulk properties of the material also affect the shrinking behavior, typical for collagen hydrogels. Shrinking is to be avoided since it would negatively impact the shape fidelity post-printing. As shown in Figure 3, for the COL2 samples a significant shrinking can be observed, in accordance with the previously morphological results and confirming that this collagen concentration has major limitations, even in the presence of TA. Instead, COL4 and COL6 showed high volume stability, with negligible effect due to the interaction with TA. Indeed, by increasing collagen concentration, the water content within the sample volume is reduced and the network density is increased. This explains both the higher rheological properties and the improved volume stability. Moreover, these results once again show that the bulk properties are not affected by TA. In this light, COL4 and COL6 are considered as promising candidates for the TA-mediated bioprinting approach. Altogether, results from SEM, FTIR, and rheology suggested that there is an interaction between the collagen and TA and that for COL4 and COL6 this interaction affects solely the outer surface of the scaffold while preserving bulk collagen organization and properties.

To further validate our findings, additional SEM images were acquired on the cross-sections to better understand the zone-dependent effect of TA on collagen. By comparing the external surface to the core (Figure 4), three zones were identified (Murphy and Atala, 2014): an outer layer (Li et al., 2016); a transition zone; and (Mota et al., 2020) a core area (Figure 4A), as can be seen for the COL4-TA10 condition. The more compact structure observed on the outer layer is comparable to the morphology shown in previous studies mixing collagen and TA (Yeo and Kim, 2017; Natarajan et al., 2013). Similar to the approach used in this study, Lee et al. reported the use of TA for external crosslinking of collagen scaffolds (Lee et al., 2018). However, in their study, the effect of TA was not investigated in terms of morphology and collagen fiber formation and thus cannot be compared. The effects of TA are independent from its concentration: indeed, increasing the TA concentration to 20 mg/mL exerts similar effects to the 10 mg/mL. As can be seen in Figure 4B, the COL4-TA20 sample shows the same organization in three zones as observed for COL4-TA10. The morphology of both the outer layer and the core were not affected by the increased TA concentration. However, prolonged incubation time has noticeable effects on the collagen hydrogels. Figure 4D shows COL4 samples incubated with TA10 and TA20 for 30 min. Following TA incubation, samples became ‘granulose’ over time (Figure 4D). This type of morphology resembles what was reported by Baldwin et al. for their collagen-TA beads prepared with 10 mg/mL of TA (Baldwin et al., 2021), despite differences in collagen content between the two studies. It is worth highlighting that in the study presented here, after 30 min of TA incubation, collagen organization into fibers was no longer observed. These results demonstrate that despite the relatively short period of exposure (30 min) TA can alter the bulk volume of the sample in which cells will be eventually embedded and can result in the presence of TA traces. Therefore, the contact time between the collagen and the TA is crucial, further supporting the proposed approach of the TA-supporting bath as a temporary means, needed exclusively during the extrusion process for collagen crosslinking to enable printability. Concerning the collagen concentration, increasing from 4 mg/mL to 6 mg/mL does not alter in a significant way the three layers organization previously described. Finally, the presence of a core zone and the thin thickness of the outer layer corroborates the rheological analysis and volume stability results, which show no effect of the TA-supporting bath on the bulk properties.

To assess the ability of the TA-mediated crosslinking to enable filament extrusion, the proposed strategy was used to 3D print single lines and the shape fidelity was evaluated. While Figure 5 certainly proves the ability of the TA bath to enable the extrusion of a collagen filament, Figures 5B, E equally prove that this mechanism is needle diameter dependent: both for COL4 and COL6, in case of a 15G needle, the interaction with TA was not sufficient to enable shape fidelity, thus resulting in unshaped constructs. Furthermore, Figure 5 shows the TA concentration-dependence of the shape fidelity: by increasing TA concentration up to 20 mg/mL, an increase in shape fidelity was observed for both COL4 (Figure 5C) and COL6 (Figure 5F). In addition, the measurement of the thickness highlighted the homogeneity of the analyzed samples (Figures 5G, H). The correlation of the shape fidelity post-printing with the TA concentration is comparable to the one previously shown by Yeo and Kim. (2017): despite in this work TA takes the role of external supporting bath, contrary to their COL-TA mixing, the observed trend is similar.

Concerning the biological outcome for the tested conditions, live/dead results post-bioprinting (Figures 6A, B) showed TA toxicity on day 1. This effect was observed for both TA concentrations tested. Baldwin et al. have already previously demonstrated a correlation between increasing TA concentration and decreasing cell attachment and viability on their COL-TA beads (Baldwin et al., 2021). However, in their approach, TA10 showed no detrimental effect on cell viability. In this study, on the contrary, both TA10 and TA20 showed comparable levels of (low) viability. It is reasonable to relate the cell mortality to the stress that cells experience during the bioprinting process, at least partially. However, Yeo et al. in their COL-TA hybrid bioink reported such levels of mortality starting at 50 mg/mL of TA, while at TA20 cell viability was around 90% (Yeo and Kim, 2017). Despite the levels of mortality at day 1 post-bioprinting, results at day 3 and day 7 showed an important improvement in cell viability, up to 80%, thus corroborating the hypothesis that the initial low viability can be attributed to the temporary contact with TA and it can be fully recovered afterward.

## 5 Conclusion

Despite their favorable biological properties, the use of collagen hydrogels in 3D bioprinting is still hampered by their poor shape retention and extrudability. In this work, the temporary use of an external bath of tannic acid has been proposed as initiator of the crosslinking of the outermost collagen layer to improve its printability. Different concentrations of both collagen (2, 4, and 6 mg/mL) and tannic acid (5, 10, and 20 mg/mL) have been explored. From the performed analyses, collagen concentrations above 4 mg/mL showed the best results in terms of rheological properties and printability. Regarding tannic acid, the tested concentrations showed how, when used as an external and temporary bath, only the external portion of the collagen is affected by tannic acid, while retaining the recognized collagen properties in the bulk. This translated into an improved printability and shape fidelity. Moreover, minimal negative effect of tannic acid on embedded cell viability was noted, especially at lower concentration. In conclusion, the presented data show how an external and temporary tannic acid bath can be used to achieve the 3D printing of pure collagen hydrogels for TERM applications. In particular, TA10 could be beneficial for the printing of cell-containing bioinks, whereas the TA20 could be optimal for cell-free collagen hydrogels. In the future, a more in-depth biological characterization, involving tissue-specific cell lines, will be needed in order to clearly elucidate the effects of the temporary exposure to tannic acid as well as validating the technique for the 3D printing of more complex geometries, both highly sought after for TERM.

## Data Availability

The original contributions presented in the study are included in the article/Supplementary Material, further inquiries can be directed to the corresponding author.
