# Supplementary material for: Enabling 3D bioprinting of cell-laden pure collagen scaffolds via tannic acid supporting bath
Source: Front Bioeng Biotechnol. 2024 Sep 4;12:1434435. doi: 10.3389/fbioe.2024.1434435 (PMC11408190; doi:10.3389/fbioe.2024.1434435)
Supplement: Supplementary file 1 [file DataSheet1.docx]

Supplementary Material

# Supplementary Figure


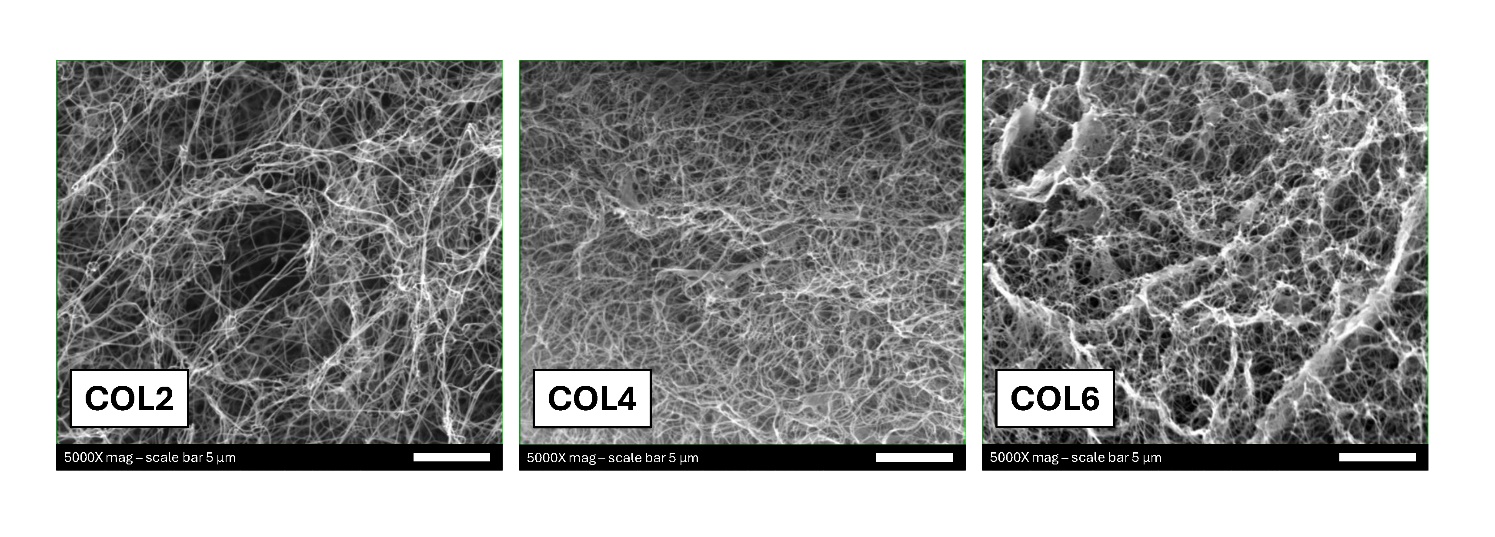
**Supplementary Figure S1.** **SEM images of collagen scaffolds at different concentrations, without TA-treatment.** COL assembles into fibers at all the studied concentrations. SEM images proved that an increase in collagen concentration is correlated with an increase in network density. Furthermore, at high collagen concentration (COL6) fiber agglomeration could be observed.
